# Homing in on Endogenous Badnaviral Elements: Development of Multiplex PCR-DGGE for Detection and Rapid Identification of Badnavirus Sequences in Yam Germplasm

**DOI:** 10.3389/fpls.2022.846989

**Published:** 2022-05-10

**Authors:** Gonçalo Silva, Moritz Bömer, Aliyu A. Turaki, Chukwuemeka K. Nkere, P. Lava Kumar, Susan E. Seal

**Affiliations:** ^1^Natural Resources Institute, University of Greenwich, Chatham Maritime, United Kingdom; ^2^Kebbi State University of Science and Technology Aliero, Birnin Kebbi, Nigeria; ^3^International Institute of Tropical Agriculture (IITA), Ibadan, Nigeria; ^4^Department of Crop Protection and Environmental Biology (CPEB), University of Ibadan, Ibadan, Nigeria; ^5^National Root Crops Research Institute (NRCRI), Umudike, Nigeria

**Keywords:** badnavirus, multiplex PCR, denaturing gradient gel electrophoresis, yam, *Dioscorea* spp., West Africa, endogenous badnaviral elements

## Abstract

Viruses of the genus *Badnavirus* (family *Caulimoviridae*) are double-stranded DNA-reverse transcribing (dsDNA-RT) plant viruses and have emerged as serious pathogens of tropical and temperate crops globally. Endogenous badnaviral sequences are found integrated in the genomes of several economically important plant species. Infection due to activation of replication-competent integrated copies of the genera *Badnavirus*, *Petuvirus* and *Cavemovirus* has been described. Such endogenous badnaviral elements pose challenges to the development of nucleic acid-based diagnostic methods for episomal virus infections and decisions on health certification for international movement of germplasm and seed. One major food security crop affected is yam (*Dioscorea* spp.). A diverse range of *Dioscorea* bacilliform viruses (DBVs), and endogenous DBV (eDBV) sequences have been found to be widespread in yams cultivated in West Africa and other parts of the world. This study outlines the development of multiplex PCR-dependent denaturing gradient gel electrophoresis (PCR-DGGE) to assist in the detection and analysis of eDBVs, through the example of analysing yam germplasm from Nigeria and Ghana. Primers targeting the three most prevalent DBV monophyletic species groups in West Africa were designed to improve DGGE resolution of complex eDBV sequence fingerprints. Multiplex PCR-DGGE with the addition of a tailor-made DGGE sequence marker enables rapid comparison of endogenous badnaviral sequence diversity across germplasm, as illustrated in this study for eDBV diversity in yam.

## Introduction

Members of the genus *Badnavirus*, family *Caulimoviridae*, are the most widespread viruses known to infect yams and other economically important crop plants, such as banana, black pepper, cacao, citrus, sugarcane, and taro globally (Borah et al., 2013; Bhat et al., 2016). Their high serological and genetic diversity challenges the development of reliable diagnostic tests for badnaviruses, as experienced for several crops, e.g., banana, sugarcane and yam (Lockhart, 1986; Harper et al., 2005; Kenyon et al., 2008; Muller et al., 2011). The development of nucleic acid-based diagnostic methods is further complicated due to the presence of integrated badnavirus sequences in some host plant genomes. The discovery of these endogenous counterparts poses serious challenges for reliable diagnosis of episomal infections, taxonomy, seed certification, safe movement of germplasm, and disease management (reviewed by Bhat et al., 2016).

The vast majority of endogenous badnaviral elements are likely to be inert and essentially signatures of viral infections that may have occurred millions of years ago. To date, only a limited number of endogenous badnaviral elements are known to be able to cause episomal systemic virus infections *de novo*, following their activation in response to abiotic stresses and other undetermined factors, including breeding (Lockhart et al., 2000; Richert-Pöggeler et al., 2003; Chabannes et al., 2013). Recent attempts to inactivate endogenous banana streak virus (eBSV) sequences in the host plantain (*Musa* spp.) genome using CRISPR/Cas9-based genome editing appear promising for overcoming the major potential limitation to breeding programmes posed by specific eBSVs which are activated by hybridization process used in crop breeding (Tripathi et al., 2019).

*Dioscorea* bacilliform viruses (DBVs) are members of the genus *Badnavirus* and are extremely diverse with at least 15 species groups recognized to date (Kenyon et al., 2008; Bousalem et al., 2009; Umber et al., 2014; Bömer et al., 2016). Yam plants hosting diverse DBVs and mixed infections thereof are a common occurrence in West African yam germplasm (Bömer et al., 2016; Umber et al., 2017), potentially leading to recombinant badnaviruses (Bömer et al., 2018a). Vegetative propagation of yam through its tubers leads to the perpetual propagation of DBVs across cultivation cycles. There is an urgent need for a sustainable supply of virus-free planting material and the scarcity of ‘clean’ (i.e., virus-free) material is one of the main factors limiting yam production in West Africa (Maroya et al., 2014; Aighewi et al., 2021). Efforts for the development of modern yam seed production methods and the establishment of sustainable seed systems are ongoing (Maroya et al., 2014; Aighewi et al., 2015; Aidoo et al., 2021), and need to be accompanied by the development of accurate diagnostic tools targeting viruses infecting yam (Silva et al., 2015, 2018; Nkere et al., 2018; Bömer et al., 2019; Umber et al., 2020).

Concerning yam badnaviruses, these diagnostic tools need to differentiate endogenous DBV (eDBV) sequences from those representing episomal particles. Endogenous DBV sequences were first discovered in yam in genomes of the *D. cayenensis-rotundata* complex (Seal et al., 2014; Umber et al., 2014). Their discovery meant that classical PCR with generic badnavirus primer pair (Badna-PCR) diagnostic techniques (Yang et al., 2003) were not suitable for the detection of episomal DBV infections, as amplification products were also generated from eDBVs. Umber et al. (2014) highlighted that the episomal or endogenous nature of a badnavirus sequence could not be determined from its phylogenetic position, as eDBVs did not form a well-defined phylogenetic group, but were found to be present in many different clades.

Considerable efforts have been made to differentiate between DBVs (Bömer et al., 2016, 2018b; Sukal et al., 2017, 2019) and eDBVs (Seal et al., 2014; Umber et al., 2014, 2017), an essential step to develop methods for virus indexing of yam germplasm prior to its multiplication. Rolling circle amplification (RCA) was found to be useful for episomal virus amplification. However, RCA has also been shown to amplify integrated sequences at low frequency and plant plastid sequences (Bömer et al., 2016) and hence sequencing is required to confirm RCA results. Immunocapture-PCR approaches have had limited success due to the lack of availability of effective antisera (Seal et al., 2014; Bömer et al., 2016). The approach of combining cloning and sequencing of PCR products using the generic badnavirus primer pair (Yang et al., 2003) has the disadvantage of potentially failing to identify all sequence diversity if clones selected are not representative of the total diversity present within a tested plant.

High-throughput sequencing (HTS) of small RNAs (sRNA) has been used to characterize endogenous sequences of *Florendovirus*, a putative new genus of the family *Caulimoviridae* (Geering et al., 2014). HTS was also used to identify badnavirus sequences integrated in the genome of cacao (*Theobroma cacao*; Muller et al., 2021) and locate the exact position of the viral insert. In yam and many other crops HTS is of limited use because the endogenous sequences described to date are numerous, fragmented and rearranged (Seal et al., 2014; Umber et al., 2014). Further the full extent of eDBVs is not known and is not possible to locate the position of viral inserts and/or the flanking host genome sequences. HTS datasets for analysis of eDBVs in yam have been challenged also by the need for extensive bioinformatics input to try to resolve complex mixtures of eDBVs.

Denaturing gradient gel electrophoresis (DGGE) is a technique that has been widely used in diversity studies of viral genomes (Short and Suttle, 1999; Lyttle et al., 2011; Carmona et al., 2012), as well as the study of plant genome polymorphisms (Riedel et al., 1990; Liu and Lowes, 2013). In DGGE, double stranded and partially denatured DNA differs in its mobility when analysed by polyacrylamide gel electrophoresis and PCR fragments can be differentiated based on single base changes through the addition of a GC clamp (Sheffield et al., 1989; Top, 1992; Green et al., 2010). PCR-dependent denaturing gradient gel electrophoresis (PCR-DGGE) for rapid indication of badnavirus sequence diversity in samples containing multiple eDBVs was first reported by Turaki et al. (2017). Phylogenetic positions of many DGGE-derived yam badnavirus/eDBV sequences suggested that many more putative badnaviruses and eDBVs remain undiscovered, but accurate resolution and further characterization was challenged by complex DGGE fingerprints (Turaki et al., 2017). The design of monophyletic group-specific primers was proposed to improve DGGE resolution by focusing the analysis on the most prevalent and interesting DBV species (Turaki et al., 2017), such as the DBV species groups K5, K8, K9 and U12 which have all been reported to also contain eDBV sequences (Seal et al., 2014; Umber et al., 2014; Turaki et al., 2017).

The aims of this study were therefore (1) to determine whether multiplex PCR-DGGE using DBV species group-specific primers alongside a tailored DGGE yam badnavirus marker would improve DGGE fingerprint resolution making it more useful for analysis of West African yam germplasm and (2) to evaluate the suitability of this method as a rapid and simple tool for identification of the most prevalent conserved eDBVs across yam germplasm.

The multiplex PCR-DGGE developed is shown to facilitate the detection of sequence diversity in badnavirus PCR products and would be an applicable method for the analysis of endogenous badnavirus sequences from any plant host, or the resolution of other complex products where HTS is not a viable option. It assists the selection of interesting target sequences and reduces the need for labour-intensive screening of redundant clones, hybridization protocols and/or bioinformatic analyses. Multiplex PCR-DGGE is cost-effective when a large number of samples need to be analysed.

## Materials and Methods

### Plant Material

Tubers of *Dioscorea rotundata* breeding lines (*n* = 13) and landraces (*n* = 3) used in this study were grown in a quarantine aphid-proof glasshouse at the Natural Resources Institute (NRI, Chatham Maritime, United Kingdom), as described by Mumford and Seal (1997). These yam tubers were originally provided by the International Institute of Tropical Agriculture (IITA, Ibadan, Nigeria). DNA/leaf specimens (*n* = 43) collected during field surveys in Nigeria and Ghana by IITA in 2013 were included in the analysis. Selected individual leaf samples were collected from each plant and placed in small polythene bags (10 cm × 15 cm) and processed immediately for total nucleic acids extraction. Clones of the same yam lines are differentiated in their names by A and B, e.g., *D*. *rotundata* accession (TDr) 89/02475-A and -B, TDr 1892-A and -B, TDr00/00403-A and -B, and *D*. *rotundata* landrace Adaka-A and -B.

### Total Nucleic Acid Extraction and Yam Badnavirus-Specific Multiplex PCR

Total nucleic acids (TNA) were extracted from fresh yam leaves (~100 mg) using a modified cetyltrimethylammonium bromide (CTAB) method adapted from Lodhi et al. (1994) and as described by Bömer et al. (2018b). This extraction method was used unless specified otherwise. TNAs were screened for badnavirus sequences by PCR as described previously (Bömer et al., 2016, 2018b; Turaki et al., 2017) using the generic badnavirus Badna-forward primer (FP) and Badna-reverse primer (RP; Yang et al., 2003), and amplifying a 579 bp region (528 bp excluding primer sequences and representing only complete amino acids) of the RT-RNaseH gene used for taxonomic assessment of badnaviruses (Teycheney et al., 2020). Both Badna-FP and Badna-RP primers were modified by the addition of a GC clamp as described previously (Turaki et al., 2017), and referred to as BF-GC (5′ CGC CCG CCG CGC GCG GCG GGC GGG GCG GGG GCA CGG GGG GAT GCC ITT YGG IIT IAA RAA YGC ICC 3′) and BR-GC (5′ CGC CCG CCG CGC GCG GCG GGC GGG GCG GGG GCA CGG GGG GCC AYT TRC AIA CIS CIC CCC AIC C 3′), respectively. Primers K8-F, K8-R, K9-R and U12-R used for multiplex Badna-PCR were designed in this study (Supplementary Table S1). Multiplex Badna-PCR amplifications for DGGE analysis were set up in 25 μl reactions containing 1 μl of template (TNA extractions diluted to 20 ng/μL), 1.2 μM of BF-GC, 0.4 μM each of the K8-R, K9-R and U12-R primers, 0.25 mM of each deoxynucleotide triphosphate (dNTP), 1 U DreamTaq DNA polymerase and 1× DreamTaq Green buffer (Thermo Scientific, United Kingdom) containing 2 mM MgCl2. The cycle conditions for multiplex Badna-PCR amplification were 95°C for 5 min, followed by 35 cycles of 94°C for 1 min, 55°C for 45 s, 72°C for 2 min and a final extension of 72°C for 10 min. Prior to DGGE analysis, PCR products were confirmed to be of the correct size by agarose gel electrophoresis through 1.5% (w/v) agarose gels including 1× SYBR Safe DNA gel stain (Thermo Fisher Scientific, United Kingdom) in 0.5× Tris-Borate-EDTA (TBE) buffer. All primers described were synthesized by Sigma-Aldrich (United Kingdom).

DNAs of a subset of yam leaf samples analysed in this study were extracted using the Plant Tissue mini protocol of the DNeasy Plant mini kit (Qiagen) following the TissueLyzer procedure according to the manufacturers’ instructions. DNAs were eluted using 50 μl of buffer AE and further diluted 1:5 with molecular grade water (Sigma, United Kingdom) prior to PCR using 1 μl of template as described before. Yam DNA samples analysed by multiplex PCR-DGGE were first screened by Badna-PCR as described by Bömer et al. (2018b) using 50 μl PCR reactions. These Badna-PCRs were further purified using the reSourceTM PCR Purification Kit (Source BioScience, United Kingdom) and then used as templates (1 μl equivalent to approx. 30 ng of PCR products) in 50 μl multiplex PCR amplifications using primers BF-GC, K8-R, K9-R and U12-R and conditions as outlined above.

### Denaturing Gradient Gel Electrophoresis

The DGGE methodology used for yam badnavirus diversity studies was modified from that of Turaki et al. (2017) as described below. DGGE was performed using the INGENYphorU-2 × 2 apparatus (INGENY, Netherlands) according to the manufacturers’ instructions and comments provided in the protocol by Green et al. (2010). Gradient gels were formed using a peristaltic pump (Rietschle Thomas, Germany) and a gradient maker device (INGENY) and contained 6.5% (v/v) polyacrylamide (37.5:1 ratio of acrylamide:bis-acrylamide; National Diagnostics, United States). The denaturing gradients of the gels were 30–55% (top to bottom) unless stated otherwise (where 100% is 7 M urea and 40% (v/v) deionized formamide) and prepared in 1× Tris-acetate-EDTA (TAE) electrophoresis buffer. A stacking gel was used for sample (20 μl) loading. Electrophoresis was performed at 80 V at a temperature of 60°C for 18 h and gels were stained with 1X SYBR Gold nucleic acid gel stain (Invitrogen, Life Technologies, UK) in 1× TAE for 30 min at room temperature and subsequently destained in deionized water. Visualization took place on a UV transilluminator (G-box Chemi HR16, Syngene, United Kingdom) and bands of interest were excised from DGGE gels using a sterile scalpel. The DNA was eluted by soaking in 100 μl of molecular grade water (Sigma, United Kingdom) at 4°C overnight. Aliquots were diluted 1:10 and re-amplified by PCR using the multiplex Badna-PCR primer combination followed by PCR purification and cloning using the pGEM-T Easy vector system (Promega, United Kingdom) according to the manufacturers’ instructions prior to sequencing with standard sequencing primers SP6 and T7. To obtain a consensus sequence and control for cross-contamination, two clones from each excised DGGE band were sequenced. All sequencing in this study was performed by Source BioScience (Nottingham, United Kingdom).

### Sequence Analysis and Phylogeny

Yam badnavirus partial RT-RNaseH nucleotide sequences were analysed using MEGA7 (Kumar et al., 2016). For this, Badna-FP/-RP and vector sequences were removed, and the edited sequences were used for similarity basic local alignment search tool (BLAST) searches in the National Centre for Biotechnology Information (NCBI) GenBank database. Multiple alignments of 320 bp long partial RT-RNaseH sequences (inside BF-GC and including the K8-R primer binding sites) were performed using the CLUSTALW default settings in MEGA7, where phylogenetic trees were created using the maximum likelihood method with the Kimura 2-parameter model (Kimura, 1980) and bootstrap values for 1,000 replicates. Sequences within the genus *Badnavirus* differing in their RT-RNaseH coding region by more than 20% (sequence divergence) meet the species demarcation criterion according to the International Committee on Taxonomy of Viruses (ICTV; Teycheney et al., 2020). Two hundred and six yam badnavirus partial RT-RNaseH sequences described by Turaki et al. (2017), and the following virus sequences were obtained from the GenBank and used for comparative analyses: banana streak OL virus (BSOLV, AJ002234); cacao swollen shoot Togo A virus (CSSToAV, AJ781003); Commelina yellow mottle virus (ComYMV, NC001343); rice tungro bacilliform virus (RTBV, X57924); sugarcane bacilliform MO virus (SCBMOV, M89923); and taro bacilliform virus (TaBV, AF357836). For consistency, the grouping system reported by Kenyon et al. (2008) was adopted in this study and is similar to Turaki et al. (2017). One group (U12) reported by Umber et al. (2014) and three groups (T13–T15) described by Bömer et al. (2016) were also added to the phylogenetic analysis.

### High-Resolution Melt Analysis

Real-time amplifications were carried out on a Bio-Rad CFX96TM Real Time System as part of the C1000 Touch Thermal Cycler (Bio-Rad Laboratories, United States) using the Precision Melt Supermix (Bio-Rad Laboratories, United States) allowing post-PCR high-resolution melt (HRM) analysis according to the manufacturer’s instructions. A single reaction with a final volume of 10 μl contained 5 μl of 2× Precision Melt Supermix, 200 nM of each primer (Badna-FP combined with K8-R, K9-R or U12-R) and 4 μl of 1:100 diluted plasmid preparations (approx. 1–3 ng/μl) of selected DGGE clones. All reactions were performed in duplicates. For thermal cycling, an initial denaturation step at 95°C for 2 min was followed by 45 cycles of 95°C denaturation for 10 s, 56°C annealing for 30 s, and extension at 72°C for 30 s with the plate read in SYBR scan mode. HRM conditions were 95°C for 30 s, followed by 60°C for 1 min and ramping from 65°C to 95°C in 0.1°C increments (10 s per step), during which the fluorescence data was collected. The HRM data was automatically analysed using the Precision Melt AnalysisTM software (Bio-Rad Laboratories, United States) with default settings, but raising the Tm difference threshold to 0.5°C.

## Results

### Design of Improved Primers Targeting Yam Badnavirus Species Group K8, K9 and U12

The majority of DBV sequences previously identified by DGGE belong to yam badnavirus monophyletic species group K8, followed by groups K9 and U12 (Turaki et al., 2017) and all three species groups have been shown to contain eDBV sequences (Umber et al., 2014). Hence, we focused our attention on these three species groups. An alignment of 204 partial RT-RNaseH sequences from GenBank belonging to group K8 and containing a mixture of episomal DBV and eDBV sequences was used to design group K8-specific forward or reverse primers, to be combined with either the Badna-FP or Badna-RP generic primer in PCR amplifications. Similarly alignments of 73 K9 and 34 U12 partial RT-RNaseH GenBank sequences were used to design reverse K9 and U12 primers (K9-R and U12-R, Supplementary Table S1) to be used in a multiplex PCR in combination with generic badnavirus and K8-specific primers.

### K8-Specific Primers Improve Separation and Reduce Complexity of DGGE Fingerprints

**Figure 1:**
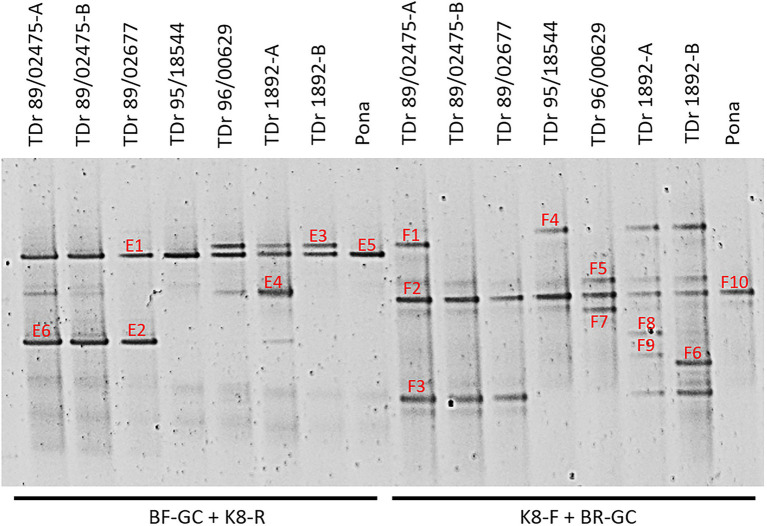
PCR-DGGE analysis of partial reverse transcriptase ribonuclease H (RT-RNaseH) badnavirus sequences from seven *Dioscorea rotundata* breeding lines (labelled TDr) and one landrace (Pona) comparing PCR amplifications using the generic badnavirus primer pair with a GC clamp fused to either the forward (BF-GC) or reverse primer (BR-GC) in combination with monophyletic species group-specific primers K8-F or K8-R. *Dioscorea rotundata* accession TDr 89/02475-A and -B and TDr 1892-A and -B are clones of the same yam accessions. Sixteen distinct bands (E1–E6; F1–10) were excised, re-amplified, cloned and sequenced. Typically, two clones per excised DGGE band were sequenced, and both sequences are presented in Table 1 except for those which were found to be 100% identical to each other.

PCR-DGGE analysis requires a GC clamp that can be added to either the forward Badna-FP or reverse Badna-RP primer. We decided to test both degenerate Badna-GC clamp primers (referred to as BF-GC or BR-GC) in combination with the newly designed non-degenerate group K8-specific primers on a selection of eight yam samples previously tested by Turaki et al. (2017). PCR amplifications using both primer combinations produced amplicons of the expected sizes and were further analysed by DGGE (Figure 1).

The sequences corresponding to six bands (E1-6) of the BF-GC and K8-R primer combination, representing four distinct band positions on the DGGE were analysed using BLAST searches, confirming their species group K8 identity. For example, sequence NGlE5Dr of band E5 was 100% identical to a known endogenous sequence of species group K8 described by Umber et al. (2014), eDBV8 S2h9Dr (KF829997; Table 1). DGGE banding position of all clone sequences corresponding to bands E1-6 were additionally tested by running them alongside the original yam samples and a K8 group sequence marker of pooled clone sequences (Supplementary Figure S1). This confirmation step was to verify that DGGE clone sequences run at the same position as their original DGGE position, which is important for the development of a reliable DGGE marker.

**Table 1:** BLAST analysis of partial RT-RNaseH sequences cloned from DGGE bands.

Plant Accession^a^	DGGE Sequence^b^	Accession	Primers^c^	Size (bp)	NCBI Nearest Match	Identity (%)	Species Group^d^
TDr 89/02677	NGbE1aDr	MK213586	BF-GC + K8-R	320	NGb58Dr (KY555554)	99	K8
TDr 89/02677	NGbE1bDr	MK213587	BF-GC + K8-R	320	NGb58Dr (KY555554)	99	K8
TDr 89/02677	NGbE2aDr	MK213588	BF-GC + K8-R	320	NGb58Dr (KY555554)	99	K8
TDr 89/02677	NGbE2bDr	MK213589	BF-GC + K8-R	320	NGb58Dr (KY555554)	100	K8
TDr 1892-B	NGbE3aDr	MK213590	BF-GC + K8-R	320	NGb23bDr (KY555499)	99	K8
TDr 1892-B	NGbE3bDr	MK213591	BF-GC + K8-R	320	NGb23bDr (KY555499)	98	K8
TDr 1892-A	NGbE4aDr	MK213592	BF-GC + K8-R	320	NGb5Dr (KY555463)	99	K8
TDr 1892-A	NGbE4bDr	MK213593	BF-GC + K8-R	320	NGb5Dr (KY555463)	99	K8
Pona	NGlE5Dr	MK213594	BF-GC + K8-R	320	eDBV8_S2h9Dr (KF829997)	100	K8
TDr 89/02475-A	NGbE6aDr	MK213595	BF-GC + K8-R	320	NGb58Dr (KY555554)	99	K8
TDr 89/02475-A	NGbE6bDr	MK213596	BF-GC + K8-R	320	NGb58Dr (KY555554)	100	K8
TDr 89/02475-A	NGbF1aDr	MK213597	K8-F + BR-GC	391	NGb8aDr (KY555468)	99	T13
TDr 89/02475-A	NGbF1bDr	MK213598	K8-F + BR-GC	391	NGb5Dr (KY555463)	99	K8
TDr 89/02475-A	NGbF2aDr	MK213599	K8-F + BR-GC	391	eDBV8_S2b10Dr (KF829984)	100	K8
TDr 89/02475-A	NGbF2bDr	MK213600	K8-F + BR-GC	391	eDBV8_S2b10Dr (KF829984)	99	K8
TDr 89/02475-A	NGbF3aDr	MK213601	K8-F + BR-GC	391	NGb47bDr (KY555541)	99	K8
TDr 89/02475-A	NGbF3bDr	MK213602	K8-F + BR-GC	391	NGb57Dr (KY555553)	99	K8
TDr 95/18544	NGbF4aDr	MK213603	K8-F + BR-GC	391	DBRTV1 (KX008596)	99	T13
TDr 95/18544	NGbF4bDr	MK213604	K8-F + BR-GC	391	eDBV8_S2b10Dr (KF829984)	99	K8
TDr 96/00629	NGbF5aDr	MK213605	K8-F + BR-GC	391	NGb5Dr (KY555463)	99	K8
TDr 96/00629	NGbF5bDr	MK213606	K8-F + BR-GC	391	NGb23bDr (KY555499)	98	K8
TDr 1892-B	NGbF6aDr	MK213607	K8-F + BR-GC	391	DBALV-[3RT] (KX008595)	99	K8
TDr 1892-B	NGbF6bDr	MK213608	K8-F + BR-GC	391	DBALV-[3RT] (KX008595)	99	K8
TDr 96/00629	NGbF7aDr	MK213609	K8-F + BR-GC	391	eDBV8_S2b10Dr (KF829984)	99	K8
TDr 96/00629	NGbF7bDr	MK213610	K8-F + BR-GC	391	NGb5Dr (KY555463)	99	K8
TDr 1892-A	NGbF8aDr	MK213611	K8-F + BR-GC	391	DBRTV1-[2RT] (KX008597)	99	T13
TDr 1892-A	NGbF8bDr	MK213612	K8-F + BR-GC	391	DBALV-[3RT] (KX008595)	99	K8
TDr 1892-A	NGbF9aDr	MK213613	K8-F + BR-GC	391	eDBV8_S2b10Dr (KF829984)	100	K8
TDr 1892-A	NGbF9bDr	MK213614	K8-F + BR-GC	391	NGb5Dr (KY555463)	99	K8
Pona	NGlF10Dr	MK213615	K8-F + BR-GC	391	eDBV8_S2b10Dr (KF829984)	99	K8
TDr 1892	NGbN1Dr	MK213616	BF-GC + K8-R	320	eDBV8_S2h9Dr (KF829997)	99	K8
TDr 1892	NGbN2Dr	MK213617	BF-GC + K8-R	320	eDBV8_S2h9Dr (KF829997)	99	K8
TDr 1892	NGbN3Dr	MK213618	BF-GC + K9-R	380	eDBV9_S1e3Dr (KF829969)	100	K9
TDr 1892	NGbN4Dr	MK213619	BF-GC + U12-R	498	eDBV12_S1a4Dr (KF829956)	99	U12
TDr 00/00403-A	NGbN5Dr	MK213620	BF-GC + K8-R	320	eDBV8_S2h9Dr (KF829997)	99	K8
TDr 00/00403-A	NGbN6Dr	MK213621	BF-GC + K8-R	320	eDBV8_S2h9Dr (KF829997)	99	K8
TDr 07/00033	NGbN7Dr	MK213622	BF-GC + K9-R	380	eDBV9_S1e3Dr (KF829969)	99	K9
TDr 00/00403-A	NGbN8Dr	MK213623	BF-GC + U12-R	498	eDBV12_S1a4Dr (KF829956)	100	U12
TDr 89/02475	NGbN9Dr	MK213624	BF-GC + K8-R	320	NGb58Dr (KY555554)	99	K8
TDr 94/01108	NGbN10Dr	MK213625	BF-GC + K8-R	320	NGb58Dr (KY555554)	100	K8
TDr 00/00362	NGbN11Dr	MK213626	BF-GC + U12-R	498	eDBV12_S2a7Dr (KF829978)	99	U12
TDr 03/00058	NGbN12Dr	MK213627	BF-GC + U12-R	498	eDBV12_S2a7Dr (KF829978)	99	U12
TDr 03/00196	NGbN13aDr	MK213628	BF-GC + K9-R	380	NGb29aDr (KY555510)	99	K9
TDr 03/00196	NGbN13bDr	MK213629	BF-GC + K9-R	380	NGb29aDr (KY555510)	99	K9
TDr 03/00058	NGbN14aDr	MK213630	BF-GC + U12-R	498	eDBV12_S2a7Dr (KF829978)	99	U12
TDr 03/00058	NGbN14bDr	MK213631	BF-GC + U12-R	498	NGb59Dr (KY555555)	99	U12
Adaka-B	NGlN15aDr	MK213632	BF-GC + U12-R	498	NGb59Dr (KY555555)	98	U12
Adaka-B	NGlN15bDr	MK213633	BF-GC + U12-R	498	NGb59Dr (KY555555)	98	U12
Adaka-B	NGlN16aDr	MK213634	BF-GC + U12-R	498	NGb59Dr (KY555555)	98	U12
Adaka-B	NGlN16bDr	MK213635	BF-GC + U12-R	498	NGb59Dr (KY555555)	98	U12
TDr 03/00196	NGbN17aDr	MK213636	BF-GC + K9-R	380	NGb29aDr (KY555510)	99	K9
TDr 03/00196	NGbN17bDr	MK213637	BF-GC + K9-R	380	NGb29aDr (KY555510)	99	K9
TDr 03/00058	NGbN18aDr	MK213638	BF-GC + U12-R	498	eDBV12_S2a9Dr (KF829980)	100	U12
TDr 03/00058	NGbN18bDr	MK213639	BF-GC + U12-R	498	eDBV12_S2h10Dr (KF829998)	99	U12
TDr 03/00058	NGbN19aDr	MK213640	BF-GC + U12-R	498	eDBV12_S2a9Dr (KF829980)	99	U12
TDr 03/00058	NGbN19bDr	MK213641	BF-GC + U12-R	497	eDBV12_S2a9Dr (KF829980)	99	U12
TDr 00/00403-A	NGbN20aDr	MK213642	BF-GC + U12-R	497	eDBV12_S1a4Dr (KF829956)	99	U12
TDr 00/00403-A	NGbN20bDr	MK213643	BF-GC + U12-R	498	eDBV12_S1a4Dr (KF829956)	99	U12
TDr 07/00033	NGbN21aDr	MK213644	BF-GC + K9-R	380	eDBV9_S1e3Dr (KF829969)	99	K9
TDr 07/00033	NGbN21bDr	MK213645	BF-GC + K9-R	380	eDBV9_S1e3Dr (KF829969)	99	K9
TDr 07/00033	NGbN22aDr	MK213646	BF-GC + K9-R	380	eDBV9_S1e3Dr (KF829969)	99	K9
TDr 07/00033	NGbN22bDr	MK213647	BF-GC + K9-R	380	eDBV9_S1e3Dr (KF829969)	99	K9
TDr 00/00403-B	NGbN23Dr	MK213648	BF-GC + K9-R	380	eDBV9_S1e3Dr (KF829969)	99	K9
TDr 00/00403-B	NGbN24aDr	MK213649	BF-GC + K9-R	380	eDBV9_S1e3Dr (KF829969)	100	K9
TDr 00/00403-B	NGbN24bDr	MK213650	BF-GC + K9-R	573	DBRTV1-[3RT] (KX008576)	92	T13
TDr 89/02475-A	NGbI1aDr	MK213651	BF-GC + K9-R	380	eDBV9_S1e3Dr (KF829969)	99	K9
TDr 89/02475-A	NGbI1bDr	MK213652	BF-GC + K9-R	380	eDBV9_S1e3Dr (KF829969)	100	K9
TDr 89/02677	NGbI2aDr	MK213653	BF-GC + K8-R	320	NGb36aDr (KY555522)	99	K9
TDr 89/02677	NGbI2bDr	MK213654	BF-GC + K8-R	320	NGb36aDr (KY555522)	99	K9
TDr 89/02677	NGbI3aDr	MK213655	BF-GC + U12-R	498	eDBV12_S1a4Dr (KF829956)	99	U12
TDr 89/02677	NGbI3bDr	MK213656	BF-GC + U12-R	498	eDBV12_S1a4Dr (KF829956)	99	U12
TDr 96/00629	NGbI4aDr	MK213657	BF-GC + K9-R	380	NGb54Dr (KY555549)	99	K9
TDr 96/00629	NGbI4bDr	MK213658	BF-GC + K9-R	380	NGb54Dr (KY555549)	99	K9
TDr 96/00629	NGbI5Dr	MK213659	BF-GC + U12-R	498	eDBV12_S2a7Dr (KF829978)	99	U12
TDr 1892-A	NGbI6aDr	MK213660	BF-GC + K9-R	380	eDBV9_S1e3Dr (KF829969)	99	K9
TDr 1892-A	NGbI6bDr	MK213661	BF-GC + K9-R	380	eDBV9_S1e3Dr (KF829969)	99	K9
Pona	NGlI7aDr	MK213662	BF-GC + U12-R	498	eDBV12_S1a4Dr (KF829956)	100	U12
Pona	NGlI7bDr	MK213663	BF-GC + U12-R	498	eDBV12_S1a4Dr (KF829956)	99	U12
TDr 04/219	NGbI8Dr	MK213664	BF-GC + K9-R	380	NGb29aDr (KY555510)	99	K9
TDr N26-1	NGfI9aDr	MK213665	BF-GC + K8-R	319	GN4Da (AM944577)	94	K8
TDr N26-1	NGfI9bDr	MK213666	BF-GC + K8-R	320	GN4Da (AM944577)	94	K8
TDc N1-4	NGfI10aDc	MK213667	BF-GC + K8-R	320	NGb36aDr (KY555522)	99	K9
TDc N1-4	NGfI10bDc	MK213668	BF-GC + K8-R	320	NGb36aDr (KY555522)	99	K9
TDa G3-6	NGfI11aDa	MK213669	BF-GC + K8-R	320	NGb36aDr (KY555522)	97	K9
TDa G3-6	NGfI11bDa	MK213670	BF-GC + K8-R	320	NGb36aDr (KY555522)	99	K9
TDr N8-29	NGfI12aDr	MK213671	BF-GC + K8-R	320	eDBV8_S2h9Dr (KF829997)	98	K8
TDr N8-29	NGfI12bDr	MK213672	BF-GC + K8-R	320	NGb5Dr (KY555463)	99	K8
TDr N8-29	NGfI13Dr	MK213673	BF-GC + K8-R	320	eDBV8_S2h9Dr (KF829997)	99	K8
TDr N26-1	NGfI14aDr	MK213674	BF-GC + K8-R	319	VU257Dp (AM072708)	94	K8
TDr N26-1	NGfI14bDr	MK213675	BF-GC + K8-R	320	NGb36aDr (KY555522)	99	K9
TDr 99/02607	NGbJ1aDr	MK213676	BF-GC + K9-R	380	eDBV9_S1e3Dr (KF829969)	99	K9
TDr 99/02607	NGbJ1bDr	MK213677	BF-GC + K9-R	380	eDBV9_S1e3Dr (KF829969)	100	K9
TDr 1892-B	NGbJ2aDr	MK213678	BF-GC + K9-R	380	eDBV9_S1e3Dr (KF829969)	100	K9
TDr 1892-B	NGbJ2bDr	MK213679	BF-GC + K9-R	380	eDBV9_S1e3Dr (KF829969)	99	K9
TDr 04/219	NGbJ3aDr	MK213680	BF-GC + K9-R	380	NGb29aDr (KY555510)	99	K9
TDr 04/219	NGbJ3bDr	MK213681	BF-GC + K9-R	380	NGb29aDr (KY555510)	99	K9
Pona	NGlJ4aDr	MK213682	BF-GC + U12-R	498	eDBV12_S1a4Dr (KF829956)	100	U12
Pona	NGlJ4bDr	MK213683	BF-GC + U12-R	498	eDBV12_S1a4Dr (KF829956)	99	U12
TDr 89/02677	NGbJ5Dr	MK213684	BF-GC + U12-R	498	eDBV12_S1a4Dr (KF829956)	99	U12
TDr N26-1	NGfK1aDr	MK213685	BF-GC + K8-R	320	VU257Dp (AM072708)	93	K8
TDr N26-1	NGfK1bDr	MK213686	BF-GC + K8-R	320	VU257Dp (AM072708)	93	K8
TDr N37-7	NGfK2Dr	MK213687	BF-GC + K9-R	380	eDBV9_S1e3Dr (KF829969)	100	K9
TDr N34-4	NGfK3aDr	MK213688	BF-GC + K8-R	320	GyM1Dt (EF466081)	92	K8
TDr N34-4	NGfK3bDr	MK213689	BF-GC + K8-R	320	NGb36aDr (KY555522)	99	K9
TDr N39-1	NGfK4Dr	MK213690	BF-GC + K8-R	320	eDBV8_S2h9Dr (KF829997)	99	K8
TDr N40-18	NGfK5aDr	MK213691	BF-GC + K9-R	380	eDBV9_S1e3Dr (KF829969)	99	K9
TDr N40-18	NGfK5bDr	MK213692	BF-GC + K9-R	380	eDBV9_S1c5Dr (KF829963)	100	K9
TDr N40-18	NGfK5cDr	MK213693	BF-GC + K8-R	320	NGb58Dr (KY555554)	99	K8
TDr N40-18	NGfK5dDr	MK213694	BF-GC + K8-R	320	NGb58Dr (KY555554)	99	K8
TDc N1-4	NGfK6aDc	MK213695	BF-GC + K9-R	380	NGb29aDr (KY555510)	99	K9
TDc N1-4	NGfK6bDc	MK213696	BF-GC + K9-R	380	NGb29aDr (KY555510)	99	K9
TDr N33-1	NGfK7Dr	MK213697	BF-GC + K9-R	380	NGb29aDr (KY555510)	99	K9
TDc N1-4	NGfK8Dc	MK213698	BF-GC + K9-R	380	eDBV9_S1e3Dr (KF829969)	100	K9
TDr N26-1	NGfK9Dr	MK213699	BF-GC + K8-R	320	NGb36aDr (KY555522)	99	K9
TDr N26-1	NGfK10Dr	MK213700	BF-GC + K9-R	380	eDBV9_S1e3Dr (KF829969)	100	K9
TDr N37-7	NGfK11aDr	MK213701	BF-GC + K9-R	380	eDBV9_S1e3Dr (KF829969)	99	K9
TDr N37-7	NGfK11bDr	MK213702	BF-GC + K9-R	380	eDBV9_S1e3Dr (KF829969)	100	K9
TDr N8-29	NGfK12aDr	MK213703	BF-GC + U12-R	498	eDBV12_S1a4Dr (KF829956)	99	U12
TDr N8-29	NGfK12bDr	MK213704	BF-GC + U12-R	498	eDBV12_S1a4Dr (KF829956)	99	U12
TDr N34-4	NGfK13Dr	MK213705	BF-GC + K9-R	380	eDBV9_S2c7Dr (KF829987)	99	K9
TDc G1-1	GHfL1aDc	MK213706	BF-GC + K9-R	380	eDBV9_S1e3Dr (KF829969)	99	K9
TDc G1-1	GHfL1bDc	MK213707	BF-GC + K9-R	380	eDBV9_S1e3Dr (KF829969)	99	K9
TDc G1-1	GHfL2Dc	MK213708	BF-GC + K9-R	380	eDBV9_S1e3Dr (KF829969)	99	K9
TDc G1-1	GHfL3aDc	MK213709	BF-GC + K9-R	380	NGb29aDr (KY555510)	99	K9
TDc G1-1	GHfL3bDc	MK213710	BF-GC + K9-R	380	NGb29aDr (KY555510)	99	K9
TDr G5-5	GHfL4aDr	MK213711	BF-GC + K9-R	379	eDBV9_S1e3Dr (KF829969)	99	K9
TDr G5-5	GHfL4bDr	MK213712	BF-GC + K9-R	379	eDBV9_S1e3Dr (KF829969)	99	K9
TDr G11-4	GHfL5aDr	MK213713	BF-GC + U12-R	498	eDBV12_S1a4Dr (KF829956)	100	U12
TDr G11-4	GHfL5bDr	MK213714	BF-GC + U12-R	498	eDBV12_S1a4Dr (KF829956)	99	U12
TDr G28-1	GHfL6aDr	MK213715	BF-GC + K9-R	380	eDBV9_S1e3Dr (KF829969)	99	K9
TDr G28-1	GHfL6bDr	MK213716	BF-GC + K9-R	380	eDBV9_S1e3Dr (KF829969)	99	K9
TDr G23-1	GHfL7aDr	MK213717	BF-GC + K9-R	380	eDBV9_S1e3Dr (KF829969)	99	K9
TDr G23-1	GHfL7bDr	MK213718	BF-GC + K9-R	380	eDBV9_S1e3Dr (KF829969)	99	K9
TDr G31-9	GHfL8aDr	MK213719	BF-GC + U12-R	498	eDBV12_S1a4Dr (KF829956)	99	U12
TDr G31-9	GHfL8bDr	MK213720	BF-GC + U12-R	498	eDBV12_S1a4Dr (KF829956)	99	U12

a *The host plants are represented by plant accession. TDa: Dioscorea alata accession; TDc: Dioscorea cayenensis accession; TDr: Dioscorea rotundata accession*.

b The DGGE clone sequences were coded as follows: the first two letters stand for the country of origin (NG = Nigeria; GH = Ghana), third letter represent sample type (‘b’ = breeding line ‘l’ = landrace, ‘f’ = farmer field), the capital letter denotes the DGGE gel; the middle number denotes the position of the excised DGGE band; the next letter denotes the clone (a = clone a and b = clone b); and the last two letters refer to the *Dioscorea* species (e.g., Dr. = *Dioscorea rotundata*).

c BF: Badna FP; BR = Badna-RP; GC: GC clamp; K8-F = group K8-specific forward primer; K8-R = group K8-specific reverse primer; K8-R, K9-R and U12-R = group K8-, K9- or U12-specific reverse primer, respectively (see Methods for details); eDBV: endogenous *Dioscorea* bacilliform viruses.

d According to phylogenetic tree (Figure 5).

Ten bands (F1-10) of the K8-F and BR-GC primer combination were also analysed. Surprisingly, three sequences corresponding to bands F1, F4 and F8 were found to be 99% identical to sequences NGb8aDr (KY555468, Turaki et al., 2017), DBRTV1 and DBRTV1-[2RT] respectively, all of which are clustering in species group T13 and described to be of episomal nature in our previous study (Bömer et al., 2016). Interestingly, the second clones of the F1, F4 and F8 bands were identified as sequences clustering in group K8, indicating that the K8-F primer is not exclusively specific to K8 target sequences, but can also anneal to T13 sequences (despite four nucleotide mismatches) and that these sequences appear to migrate in the same position or at least in very close proximity on the DGGE. Moreover, several sequences were found to be 99% identical to the episomal K8 isolate DBALV-[3RT] (KX008595, Turaki et al., 2017). Further evaluation showed better reproducibility of biological replicates and reduced complexity using the BF-GC/K8-R primer combination in comparison to the K8-F/BR-GC primer combination. Hence, the BF-GC/K8-R primer set was used for the multiplex PCR-DGGE analysis.

### Multiplexing for Analysis of Species Groups K8, K9 and U12

**Figure 2:**
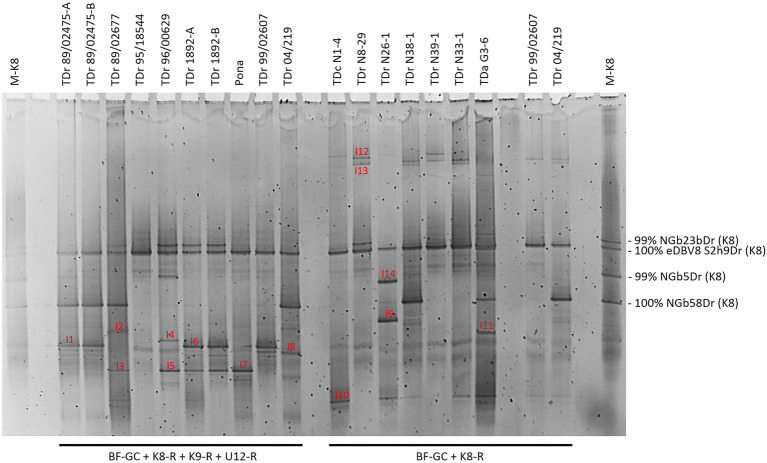
Comparison of multiplex PCR-DGGE (left-hand side) to group K8 single plex PCR-DGGE analysis (right-hand side) of partial RT-RNaseH badnavirus sequences. Samples shows are from one landrace (Pona), nine breeding lines of *D. rotundata* (TDr) and seven yam field samples from Nigeria and Ghana (TDr or TDa with sample number prefixed with N or G to denote country. Multiplex PCR amplifications were done using primers BF-GC, K8-R, K9-R and U12-R and single plex PCR with primers BF-GC and K8-R. *D. rotundata* accession TDr 89/02475-A and -B and TDr 1892-A and -B are clones of the same yam accessions. *D. rotundata* breeding lines TDr 99/02607 and TDr 04/219 were included for comparison. Fourteen distinct bands (I1–I14) were excised, re-amplified, cloned and sequenced. Typically, two clones per excised DGGE band were sequenced, and both sequences are presented in Table 1 except for those which were found to be 100% identical to each other. M-K8 denotes DGGE sequence marker (see section “Development of DGGE Marker for Quick Identification of Common Endogenous Badnavirus Sequences in Yam Germplasm”).

The multiplex PCR-DGGE analysis approach was first tested on seven samples of *D. rotundata* breeding lines and one landrace. These samples were run alongside K8 single plex PCR-DGGE analysis of seven yam field samples from Nigeria and Ghana (Figure 2). K9 and U12 single plex PCR-DGGE analysis (Supplementary Figure S2) were performed on the same yam samples shown in Figure 1. *D. rotundata* breeding lines TDr 99/02607 and TDr 04/219 were included in all four primer combinations for comparison. A total of 19 distinct bands (I1-14, Figure 2 and J1-5, Supplementary Figure S2) generated across the four primer combinations assessed by DGGE were excised, cloned and sequenced (Table 1).

Using the primer combination of BF-GC + K9-R, sequences NGbJ1a/bDr and NGbJ2a/bDr were found to be 100% identical to eDBV9 S1e3Dr (KF829969, Umber et al., 2014). This integrated badnavirus sequence appears to be present in *D. rotundata* breeding lines (e.g., TDr 99/02607 and TDr 89/02475) and landrace (e.g., TDr 1892) according to the band position (Supplementary Figure S2). This was confirmed in the multiplex PCR-DGGE (Figure 2) by sequence of NGbI1bDr from TDr 89/02475. Sequences 99–100% identical to eDBV12 S1a4Dr (KF829956, Umber et al., 2014) were identified in landraces (e.g., TDr Pona and TDr 1892) and breeding lines (e.g., TDr 89/02677 and TDr 96/00629) following PCR-DGGE using the BF-GC + U12-R primer pair.

The banding pattern detected in multiplex PCR-DGGE (Figure 2) showed an overlay with the single plex PCR-DGGEs using K8-, K9- or U12-specific primers (Figure 1 and Supplementary Figure S2). This confirms the reliability and effectiveness of the multiplex approach and thus enables focusing on species groups K8, K9 and U12 and in particular endogenous sequences within these groups in the analysis of yam germplasm by DGGE.

**Figure 3:**
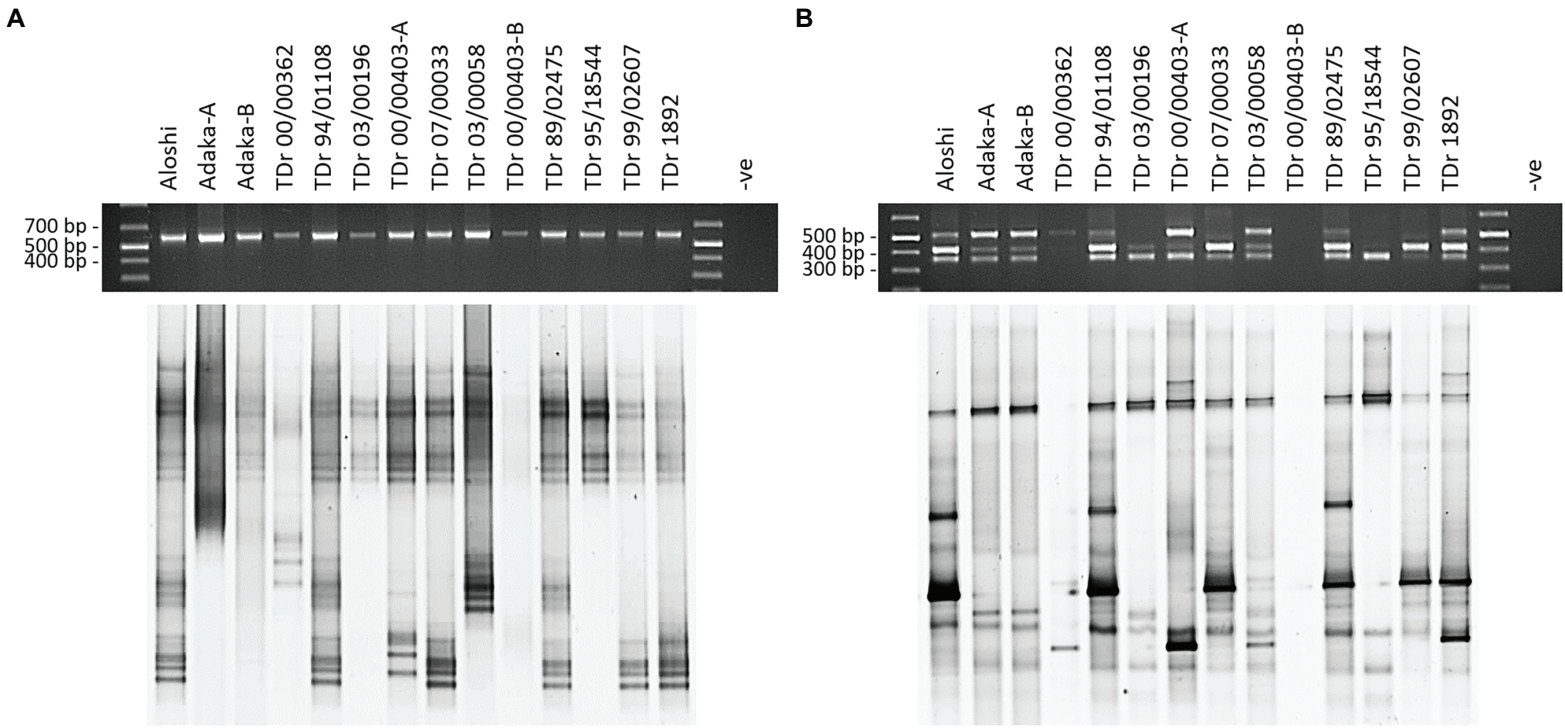
Comparison of PCR and DGGE analysis of partial RT-RNaseH badnavirus sequences amplified by Badna-PCR versus species-specific group multiplex Badna-PCR. Samples shown represent two landraces (Aloshi and Adaka) and ten breeding lines of *D. rotundata* (TDr) amplified by Badna-PCR **(A)** or species-specific group multiplex Badna-PCR **(B)**. Badna-PCRs **(A)** used BF-GC and Badna-RP primers, whereas multiplex PCR amplifications **(B)** used primers BF-GC, K8-R, K9-R and U12-R producing expected amplicon sizes of 619 bp (Badna), 387 bp (K8), 447 bp (K9) and 565 bp (U12) on a 1.5% (w/v) agarose gel (black gels on top) before DGGE loading (bottom gels). The denaturing gradient was 35–50% and DGGE was performed at 80 V at a temperature of 60°C for 18 h. Samples TDr 00/00403-A and -B and Adaka-A and -B are clones of the same yam accessions. -ve: non-template control of the PCR.

The design of monophyletic group-specific primers and their use in multiplex Badna-PCRs in this study was proposed to improve DGGE resolution while reducing the complexity of the DBV fingerprints at the same time. The successful implementation of this approach can be observed in a direct comparison of Badna-PCR and multiplex Badna-PCR products of three *D. rotundata* landraces and eleven *D. rotundata* breeding lines by DGGE (Figure 3).

### DBV Sequence Diversity in Yam Germplasm From Fields in Nigeria and Ghana

**Figure 4:**
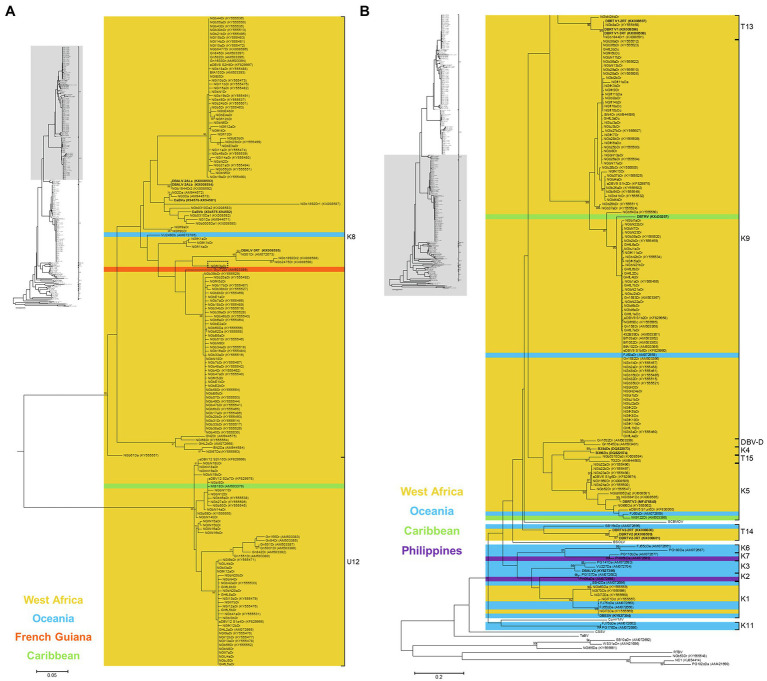
Bootstrap consensus phylogenetic tree using Maximum Likelihood method built from 320 bp long partial RT-RNaseH nucleotide sequences of 135 yam badnavirus sequences determined in this study. Included in the analysis are partial RT-RNaseH sequences with names and GenBank accession numbers of previously analysed yam samples (Eni et al., 2008; Kenyon et al., 2008; Bousalem et al., 2009; Seal et al., 2014; Umber et al., 2014, 2017; Bömer et al., 2016; Sukal et al., 2017; Turaki et al., 2017). Equivalent sequences from cacao swollen shoot Togo A virus (CSSToAV, AJ781003), banana streak OL virus (BSOLV, AJ002234), Commelina yellow mottle virus (ComYMV, NC001343), sugarcane bacilliform MO virus (SCBMOV, M89923), taro bacilliform virus (TaBV, AF357836) and outgroup rice tungro bacilliform virus (RTBV, X57924) were added, as well as representative sequences of all monophyletic groups described by Bousalem et al. (2009; where DBV-D: dioscorea bacilliform virus D), by Umber et al. (2014; U12), by Kenyon et al. (2008; K1–K11) and by Bömer et al. (2016; T13-T15). Sequences depicted in bold represent partial RT-RNaseH sequences of characterised episomal full-length DBV genomes currently available in GenBank. Coloured region/country of origin codes for the badnavirus sequence containing yam samples are provided. Sequence NGfK3aDr is highlighted in a dashed box. The phylogenetic tree was divided into sub-groups with groups K8 and U12 presented in **(A)** and all other species groups as well as the outgroups shown in **(B)**. Bootstrap values for 1,000 replicates are given when above 70%. The scale bars show the number of substitutions per base position.

A set of 43 yam samples collected from farmer fields in Nigeria and Ghana were analysed for badnavirus diversity by multiplex PCR-DGGE (Supplementary Figure S3). Detailed sample information including multiplex Badna-PCR screening results are provided in Supplementary Table S2. All 43 samples were indexed as badnavirus positive for at least one of the three species groups tested by multiplex Badna-PCR. Twenty-two out of 43 samples (51%) were scored positive for K8 sequences, whereas 58 and 67% of all 43 samples found to contain K9 and U12 sequences, respectively. Interestingly, 77% of the 22 Nigerian yam samples were indexed as K8 positive, but only 24% of the 21 Ghanaian samples appeared to have K8 DBV sequences. A greater number (64%) of Nigerian samples also scored positive for K9 sequences compared with 52% of samples from Ghana. In contrast, Ghanaian samples had more U12 sequences (71% compared to 64%; Supplementary Table S2). Use of the multiplex Badna-PCR approach allowed rapid evaluation of badnavirus/eDBV diversity. Interesting bands can be sequenced, as illustrated by the discovery of sequence NGfK3aDr, which has a unique position in the phylogenetic tree (Figure 4) and was found to have only 92% sequence similarity to GyM1Dt (EF466081, Bousalem et al., 2009) as nearest match.

The multiplex PCR-DGGE approach also highlighted unique band patterns for *D. dumetorum* cv. Una (sample N12-31), an unknown *D. cayenensis* cv. (sample N1-4), *D. rotundata* cv. Fasktse, Hembakwase, Lansirin, Alasini and Amula (samples N30-22, N26-1, N37-7, N39-1 and N40-18, respectively), and unknown *D. rotundata* varieties (samples N9-8 and N38-1) all sampled in Nigeria (Supplementary Figure S3A). The Nigerian samples appear to contain more integrated DBV sequences belonging to group K8, considering the number of bands detected by multiplex PCR-DGGE compared to the samples from Ghana, and reflect the multiplex-PCR results described in Supplementary Table S2.

### Phylogenetic Diversity of Dioscorea Badnavirus Sequences Analysed by Multiplex PCR-DGGE

All 135 partial RT-RNaseH sequences generated in this study were subjected to similarity BLAST searches in the NCBI GenBank databases and nearest matches with percent identities are presented in Table 1. Phylogenetic analysis was performed with all sequences except those 19 sequences which were produced using the K8-F and BR-GC primer combination. The remaining 116 partial RT-RNaseH sequences were trimmed to 320 bp, aligned and a phylogenetic tree created (Figure 4). Phylogenetic analyses of badnaviruses are typically based on 528 bp partial RT-RNaseH sequences inside the Badna-FP/-RP binding sites. Therefore, we needed to confirm that the shorter 320 bp DBV sequences were still targeting a long enough sequence stretch of the partial RT-RNaseH region enabling taxonomic assessment of badnaviruses (Teycheney et al., 2020). For this, a phylogenetic tree using the same badnavirus sequences as described in Bömer et al. (2016) but shortening them to the described 320 bp sequence stretch was created, and the topology of that tree was compared to the original tree based on the 528 bp long DBV sequences [Figure 2 in Bömer et al. (2016)]. Both phylogenetic trees showed very similar topologies with all DBV sequences falling into their designated species groups, confirming that the 320 bp long partial RT-RNaseH sequence stretch correlates sufficiently well for yam badnavirus phylogenetic analysis (data not shown).

The 116 partial RT-RNaseH sequences fall within four monophyletic groups (Figure 4 and Table 1) according to the suggested classification of yam badnaviruses (Kenyon et al., 2008; Bousalem et al., 2009; Umber et al., 2014; Bömer et al., 2016). Twenty-nine DBV sequences from *D. rotundata* samples clustered into monophyletic group K8, with 23 of these sharing 98–100% sequence identity to either eDBV8 clone S2h9Dr (KF829997, Umber et al., 2014) or sequences NGb58Dr, NGb23bDr and NGb5Dr detected by DGGE and suspected to be of endogenous nature as described previously (Turaki et al., 2017). These 23 sequences scatter across two highly conserved sub-groups within K8 and it appears likely that their corresponding bands represent integrated sequences, as the presence of such high identity episomal viruses in all the different yam material would not be expected. In contrast, the remaining K8 sequences (NGfI9a/bDr, NGfI14aDr, NGfK1a/bDr and NGfK3aDr) derive from field survey samples, have unique positions in the phylogenetic tree and sequence similarities of only 92–94% to their respective nearest match and are likely to therefore represent episomal DBVs.

Fifty-six DBV sequences from *D. alata*, *D. cayenensis* and *D. rotundata* samples clustered into two conserved sub-groups within monophyletic group K9 (Figure 4). Thirty-one of these shared 99–100% sequence identity to eDBV9 clone S1e3Dr (KF829969, Umber et al., 2014), 12 sequences were 99% identical to NGb29aDr (KY555510, [32]), nine sequences had their nearest match (97–99% identity) to NGb36aDr (KY555522, Turaki et al., 2017), and NGbI4a/bDr, NGfK5bDr and NGfK13Dr were identified to be very similar (99–100% identity) to NGb54Dr (KY555549, Turaki et al., 2017), eDBV9 clone S1c5Dr (KF829963, Umber et al., 2014) and eDBV9 clone S2c7Dr (KF829987, Umber et al., 2014), respectively (Table 1). Therefore, all 56 K9 sequences identified in this study appear to be eDBVs. A further 30 sequences from *D. rotundata* samples fall into monophyletic group U12 of which 25 shared 99–100% sequence identity to four known eDBV9 clone sequences described by Umber et al. (2014). The remaining five U12 sequences were 98–99% identical to NGb59Dr (KY555555, Turaki et al., 2017). The latter is 99% identical to eDBV12 clone S2h10Dr (KF829998, Umber et al., 2014). Accordingly, all 30 U12 sequences identified in this study are likely to be eDBVs. The multiplex PCR-DGGE approach using the BF-GC primer in combination with the three reverse primers specific to group K8, K9 or U12 resulted in the identification of only one sequence, sequence NGbN24bDr, which fell into an additional DBV species group (T13) described by Bömer et al. (2016) and was 92% identical to episomal DBV genome sequence DBRTV1-[3RT] (KX008576, Bömer et al., 2016).

Colour codes showing regions or countries of origin of the samples from which sequences were obtained were added to the phylogenetic tree (Figure 4), with the aim to identify DBV species groups that appear to be specific to yam growing countries in West Africa. No DBV sequences from monophyletic groups K2, K3, K6, K7 and K11 were detected to date in West African germplasm. In contrast, yam badnavirus sequences belonging to group DBV-D, K4, T13 and T15 were only found in yam samples from West Africa. DBV species groups K1, K5, K8, K9 and U12 appear to be globally distributed.

### Development of DGGE Marker for Quick Identification of Common Endogenous Badnavirus Sequences in Yam Germplasm

**Figure 5:**
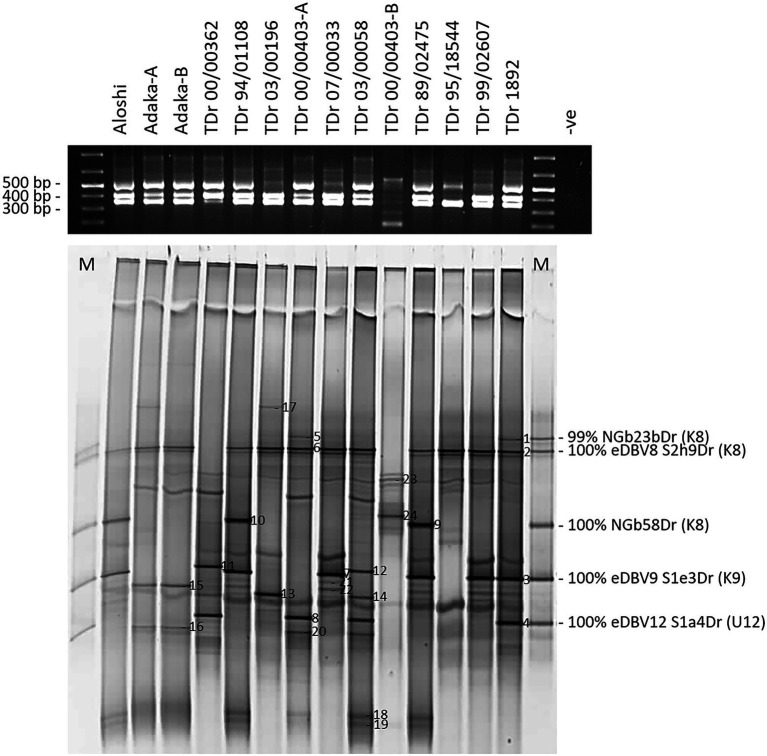
Multiplex PCR-DGGE analysis of partial RT-RNaseH badnavirus sequences from two landraces (Aloshi and Adaka-A and -B) and ten breeding lines (TDr) of *D. rotundata*. Multiplex PCR amplifications using primers BF-GC, K8-R, K9-R and U12-R were checked for their expected amplicon sizes of 387 bp (K8), 447 bp (K9) and 565 bp (U12) on a 1.5% (w/v) agarose gel (black gel photograph on top) before DGGE analysis (bottom gel). The denaturing gradient was 35–50% and DGGE was performed at 80 V at a temperature of 60°C for 18 h. M denotes DGGE sequence marker and closest NCBI BLAST identity search results are indicated including monophyletic species group assignments K8 and K9 described by Kenyon et al. (2008) and U12 by Umber et al. (2014). Band numbers 1–24 were excised, reamplified and cloned. The corresponding DGGE sequences N1-24 are presented in Table 1. *D. rotundata* accession TDr 00/00403-A and -B and landrace Adaka-A and -B are clones of the same yam accessions, but that have been grown separately for several years.

Rapid identification of conserved integrated yam badnavirus sequences by DGGE is assisted by the development of a tailored DGGE yam badnavirus marker. Following the promising approach of the K8 group sequence marker using pooled clone sequences described above, five clone sequences were selected representing the most abundant and highly conserved DBV sequences identified during this study and thus likely to represent integrated badnavirus sequences commonly found in yam germplasm, with a focus on *D. rotundata* breeding lines and landraces. Sequences NGbE3aDr, NGlE5Dr, NGbE2bDr, NGbJ1bDr and NGlJ4aDr (Table 1) were selected, their clones were pooled and run alongside 12 *D. rotundata* samples analysed by multiplex PCR-DGGE (Figure 5).

A total of 24 bands were selected as of interest, excised and sequenced (Figure 5; Table 1), and all sequences derived showed very high nucleotide identities with previously described eDBVs (Umber et al., 2014) or sequences suggested to be of endogenous nature (Turaki et al., 2017). Sequences NGbN3Dr and NGbN4Dr, for example, confirmed the presence of eDBV9 S1e3Dr (100% identical) and eDBV12 S1a4Dr (99% identical), respectively, in the TDr 1892 accession and aligned perfectly with the corresponding sequences of the DGGE marker. Unexpectedly, sample TDr 00/00403-A and TDr 00/00403-B showed a very different multiplex Badna-PCR and DGGE fingerprint. This suggests the likely possibility of mislabeling of a genetically distinct clone or other reason. Genotyping of these samples is necessary to resolve this issue. This finding demonstrates the additional value of this approach in comparing the host genomes at the same time. Sequence NGbN24bDr (also described above) derived from TDr 00/00403-B and was the only identified species group T13 sequence. Future work might resolve these unexpected findings.

The resolution of the multiplex PCR-DGGE was further improved using purified Badna-PCRs as templates for the GC clamp amplifications (Figure 3) as outlined in the Materials and Methods section. This resulted in very sharp and clear DGGE bands of similar intensities, also solving the problem of unequal loading compared with the multiplex PCR-DGGE analysis (Figures 3A,B).

**Figure 6:**
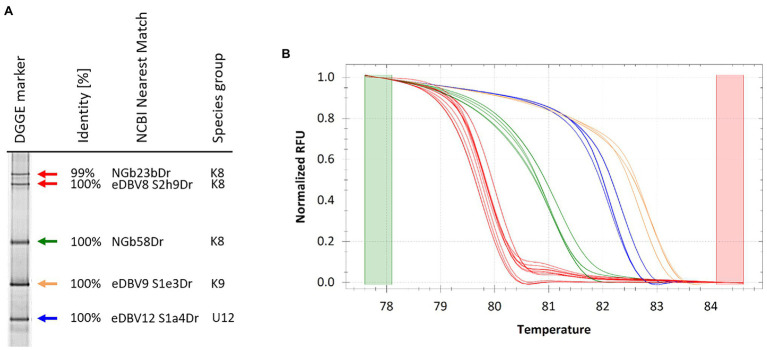
DGGE marker for quick identification of endogenous badnavirus sequences in yam germplasm and high-resolution melt (HRM) verification of amplification products following qPCR. **(A)** DGGE sequence marker extracted from DGGE analysis presented in Figure 2 and closest NCBI BLAST identity search results are indicated including monophyletic species group assignments K8 and K9 described by Kenyon et al. (2008) and U12 by Umber et al. (2014). Colour coded arrows correspond to normalized melt curves of HRM analysis shown in **(B)**. Several cloned DGGE band sequences were verified by HRM (Supplementary Table S3) following qPCR amplification confirming sequence identification based on the band position compared to the DGGE marker (Figure 2) and the sequencing results (Table 1). Melt curves of same colour indicate near identical sequences clustering together in HRM analysis.

The DGGE marker was used for quick identification of endogenous badnavirus sequences in yam germplasm and high-resolution melt (HRM) analysis was tested for verification purposes (Figure 6). Ten DGGE clone sequences corresponding to bands N1-10 (Figure 5) were selected together with the DGGE marker clone sequences and qPCR amplifications were set up in duplicates. Precision Melt Analysis^™^ software (Biorad) was used for the HRM analysis and placed all tested qPCR products into four different clusters with high percent confidence values (Supplementary Table S3) and according to their DGGE band positions compared with the DGGE marker (Figure 5). Cluster four contained the species group U9 sequences (NGbJ1bDr, NGbN3Dr and NGbN7Dr) found to be 99–100% identical to eDBV9 S1e3Dr (KF829969, Umber et al., 2014). These sequences had melting temperatures ranging from 82.7 to 82.9°C (Figure 6). The U12 sequences (NGlJ4aDr, NGbN4Dr and NGbN8Dr), 99–100% identical to eDBV12 S1a4Dr (KF829956, Umber et al., 2014), fell into HRM cluster three. These sequences were the longest amplicons (565 bp) in the multiplex Badna-PCR and run the furthest on the DGGE, but interestingly only had the second highest melting temperatures (82.2°C for NGlJ4aDr), which can be explained by a lower GC content compared with the K9 sequences. The K8 sequences NGbE2bDr, NGbN9Dr and NGbN10Dr were in HRM cluster two, with melting temperatures ranging from 81.0 to 81.3°C. Cluster one contained all remaining K8 sequences analysed by HRM. Although DGGE marker sequences NGbE3aDr and NGlE5Dr showed distinct positions on the DGGE, their melting temperatures of 79.8°C and their GC content of 39% were identified to be identical. These two sequences have only 6 bp difference (over the length of 320 bp excluding the primers) between them and were not distinguishable by HRM. In summary, HRM proved to be a valuable tool to confirm selected DBV sequences identified as interesting through comparison with the DGGE marker.

## Discussion

This study is the first to demonstrate the application of multiplex PCR-DGGE for screening for endogenous viral elements in plant genomes through the example of West African yams. Endogenous badnaviral elements define a unique and important class of dispersed repetitive DNA in plants and their recurrent invasion can contribute significantly to plant genome evolution and badnavirus resistance. An in-depth study of eBSVs in the genomes of *Musa* spp. improved our understanding of the evolution of integrated badnavirus copies (Gayral and Iskra-Caruana, 2009), and the important role of eBSVs as a reservoir protecting viral populations from local extinction (Gayral et al., 2010). Further characterization of eDBVs in yam is likely to assist in determining their role in the yam-DBV interaction as well as assisting in unravelling the evolution of yam genomes.

Access to the *D. rotundata* (Tamiru et al., 2017) and *D. alata* genomes (Bredeson et al., 2021) published in recent years will facilitate the study of eDBVs in yam, their potential allelic structures, and the dating of eDBV insertion events, as discussed by Umber et al. (2014). To date, it cannot be ruled out that infectious eDBVs exist in yam genomes and access to increasing numbers of complete annotated yam genomes will hopefully shed light into the possible impact of eDBVs on yam breeding and multiplication programmes. It is in this context where we see the strength of PCR-DGGE analysis in unravelling complex badnavirus sequences and the identification of candidate eDBV sequences. We hypothesize that multiplex PCR-DGGE targeting yam germplasm using DBV species-specific primers and a tailored DGGE sequence marker is not only a powerful new tool enabling simple, cost-effective and rapid identification of eDBV sequences but is also widely transferable to other virus genera having integrated copies in their plant host genomes and enables the comparison of the genetic host background at the same time. Accurate characterization of both episomal and endogenous badnavirus sequences is an essential prerequisite to enable meaningful studies on the biological and epidemiological significance of endogenous badnaviral elements with challenges in their differentiation hampering research to determine the significance of badnavirus infections for many important plant hosts.

### Development of Multiplex PCR-DGGE for Characterizing Badnavirus Sequences in Yam

The multiplex PCR-DGGE approach reduced the complexity of single plex DGGE fingerprints obtained previously (Turaki et al., 2017) through focusing the analysis on the most prevalent and interesting DBV species groups. The first of three DBV species groups targeted was K8, the most diverse DBV species group and possibly the most important with the largest number of publicly deposited sequences, containing a mixture of partial RT-RNaseH sequences (Kenyon et al., 2008; Bousalem et al., 2009), episomal full-length genome sequences (Briddon et al., 1999; Bömer et al., 2016), as well eDBV sequences (Umber et al., 2014). The second and third most well described DBV species groups known to contain eDBV sequences (Umber et al., 2014) were K9 and U12. Species-specific reverse primers targeting conserved regions were designed for both groups and tested positively both in single plex (Supplementary Figure S2) and multiplex (Figure 2) PCR-DGGE runs. The direct comparison of Badna-PCR and multiplex Badna-PCR products by DGGE (Figure 3) proved the significant improvement of the novel multiplex PCR-DGGE approach over the previously reported method (Turaki et al., 2017).

The improved methodology provides PCR-DGGE fingerprints that assist in the identification of conserved eDBV sequences as well as novel episomal badnaviruses or other badnavirus sequence targets of interest. In this study, PCR-DGGE fingerprinting revealed significant differences in the types of badnavirus sequences present across the West African yam samples tested and enabled the relatively rapid identification of highly conserved eDBVs present across germplasm as well as the identification of putative novel episomal badnaviruses (e.g., NGfK3aDr, Figure 2). The PCR-DGGE fingerprints provided confidence that sequences obtained were providing an accurate picture of the diversity present in samples.

Another aim of this study was the development of a tailored DGGE yam badnavirus marker for quick identification of endogenous yam badnavirus sequences by DGGE, and comparison between gels. For this, a pooled marker of five clone sequences representative of the most abundant K8, K9 and U12 sequences was generated. DGGE fingerprints from samples strongly suggest that the clones selected were representative of integrated badnavirus sequences that are widespread in West African yam germplasm. HRM analysis provided further verification of DGGE clone sequences (Figure 6) and was valuable for confirmation and grouping of selected clones with DGGE. HRM analysis could be used for clustering diversity purposes potentially replacing the need for sequencing DGGE clones.

### DBV Sequence Diversity and eDBV Phylogeny

Phylogenetic analysis the 116 partial RT-RNaseH generated sequences highlighted many badnavirus sequences that were either directly clustering with previously described eDBVs or were found to be highly conserved across a variety of yam plants tested. This suggests that it may be possible to correlate the episomal or endogenous nature of a DBV sequence to its phylogenetic position, as different (putative) eDBV sequences form well-defined phylogenetic sub-groups. Out of the 116 partial RT-RNaseH sequences, 29 were identified to belong to species group K8, of which 23 sequences were placed in two very conserved sub-groups (Figure 4) within group K8. The first of the two sub-groups was described as 8b by Umber et al. (2014), containing eDBV8 clone S2h9Dr and NGb23bDr represented in the DGGE marker (Figure 6), whereas the second sub-group around the DGGE marker sequence NGb58Dr was previously suggested to only contain eDBVs (Turaki et al., 2017), hence indicating a strong correlation between the phylogenetic position of these sequences and their suggested endogenous nature. The unique phylogenetic positions of the remaining K8 sequences, all derived from field survey samples, support their potentially episomal nature. All 56 DBV sequences belonging to species group K9 defined by Kenyon et al. (2008), clustered into two very conserved sub-groups (Figure 4). The first of the two sub-groups was described as 9b by Umber et al. (2014), containing eDBV9 clone S1e3Dr, also part of the DGGE marker (Figure 6). The second sub-group contains sequences NGb29aDr and NGb36aDr previously detected by DGGE (Turaki et al., 2017), and is referred to as 9a by Umber et al. (2014). Accordingly, all K9 sequences detected in this study are expected to be of endogenous nature based on their phylogenetic positions. Similarly, all 30 sequences falling into species group U12 defined by Umber et al. (2014) are likely to be of endogenous nature, as they cluster into two highly conserved sub-groups and share high nucleotide similarities with previously described eDBV12 sequences.

Previous studies on yam showed that eDBV sequences have undergone rearrangement resulting in mosaic structures that are typical of endogenous badnavirus elements (Umber et al., 2014). The presence of putative eDBV sequences, potentially as multiple copies, in so many of the yam lines tested (in particular *D. rotundata* germplasm), suggests that these eDBVs may confer protection from infection by related viruses *via* RNA interference as previously hypothesized for other endogenous viral elements (Harper et al., 2002; Staginnus and Richert-Pöggeler, 2006; Noreen et al., 2007; Lyttle et al., 2011; Geering et al., 2014; Umber et al., 2017). Conversely, previous research on banana demonstrated that host genomes can contain a diverse array of endogenous badnaviruses (Geering et al., 2005), of which some can undergo recombination, initiating infection *de novo* upon activation triggered by tissue culture propagation (Ndowora et al., 1999). Further research into eBSVs provided evidence that the proliferation stage of the micropropagation procedure activates the eBSV expression and episomal replication (Dallot et al., 2001). Wounding and various abiotic stress factors are also reported to activate chromosomal virus sequences (Lockhart et al., 2000; Richert-Pöggeler et al., 2003) and probably connected to weakened epigenetic control under all these conditions (Staginnus and Richert-Pöggeler, 2006). Biotechnological tools like tissue culture become ever more important and micropropagation has the potential to improve the slow vegetative propagation ratio of yam. Hence, the study of eDBVs and the screening of yam germplasm for these sequences is of paramount importance when placing *Dioscorea* species in tissue culture or under forms of abiotic or genomic stress (Bömer et al., 2019).

Tissue culture currently forms an essential part of seed yam multiplication systems and exchange of yam germplasm (Aighewi et al., 2015; Aidoo et al., 2021). The multiplex PCR-DGGE technique described will assist generating more detailed knowledge of badnavirus sequences in yam and enable the risk of a potential activation of replication-competent eDBVs to be assessed. Such knowledge will assist decision-making in national distribution of planting material as well as international exchange of yam germplasm focused on addressing improved food security for this important staple crop.

## Data Availability Statement

The datasets presented in this study can be found in online repositories. The names of the repository/repositories and accession number(s) can be found in the article/Supplementary Material.

## Author Contributions

MB, GS, AT, and SS conceived and designed the experiments. MB, AT, and CN performed the experiments. MB, AT, CN, GS, PK, and SS analysed the data. GS, MB, AT, and SS drafted the manuscript. All authors contributed to the article and approved the submitted version.

## Funding

This research was funded by the Bill & Melinda Gates Foundation (BMGF) under the “Development of On-farm Robust Diagnostic Toolkits for Yam Viruses” grant and “Enabling Research Tools for Cassava and Yam Virologists and Breeders” (Investment ID OPP1149777) to the NRI. Research work on yams at IITA is supported by the CGIAR Research Program on Roots, Tubers and Bananas (CRP-RTB) and Yam Improvement for Income and Food Security in West Africa (YIIFSWA). Funding to support open access was provided by the BMGF.

## Conflict of Interest

The authors declare that the research was conducted in the absence of any commercial or financial relationships that could be construed as a potential conflict of interest.

## Publisher’s Note

All claims expressed in this article are solely those of the authors and do not necessarily represent those of their affiliated organizations, or those of the publisher, the editors and the reviewers. Any product that may be evaluated in this article, or claim that may be made by its manufacturer, is not guaranteed or endorsed by the publisher.
