# Supplementary material for: Homing in on Endogenous Badnaviral Elements: Development of Multiplex PCR-DGGE for Detection and Rapid Identification of Badnavirus Sequences in Yam Germplasm
Source: Front Plant Sci. 2022 May 10;13:846989. doi: 10.3389/fpls.2022.846989 (PMC9127665; doi:10.3389/fpls.2022.846989)
Supplement: Supplementary file 1 [file Data_Sheet_1.docx]

Supplementary Material

# Supplementary Figures and Tables

## Supplementary Figures

**
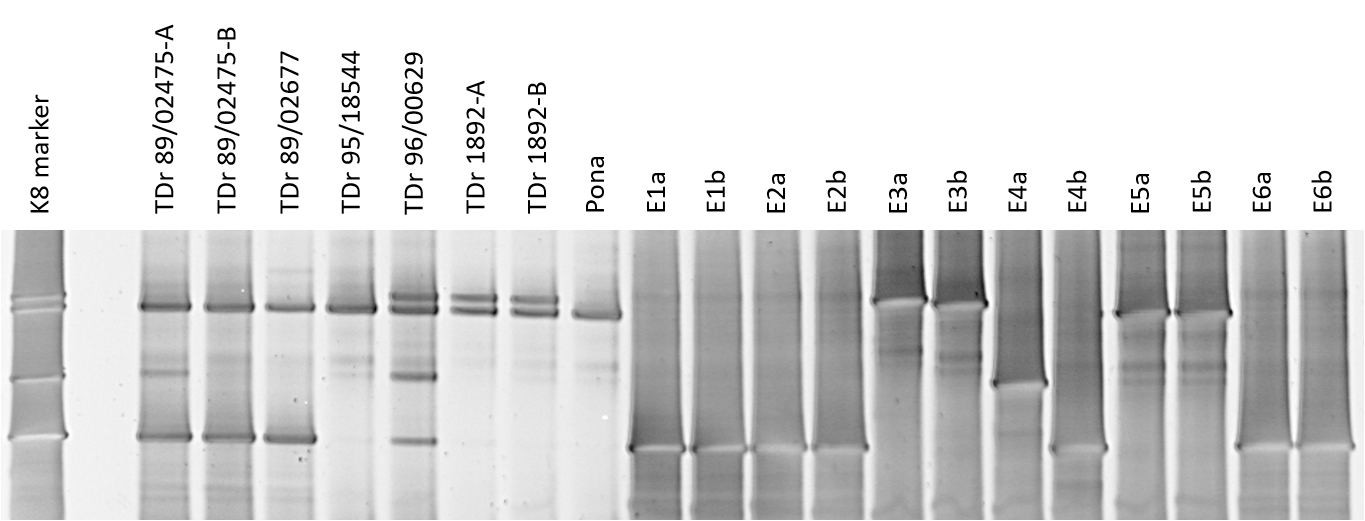
**

**Supplementary Figure 1.** K8 marker development for PCR-DGGE. PCR-DGGE analysis of all clone sequences corresponding to bands E1-6 (Figure 1) alongside the original yam samples (seven *Dioscorea rotundata* breeding lines (TDr) and one landrace Pona) and a K8 group sequence marker of pooled clone sequences. *Dioscorea rotundata* accession TDr 89/02475-A and -B and TDr 1892-A and -B are clones of the same yam accessions.

**
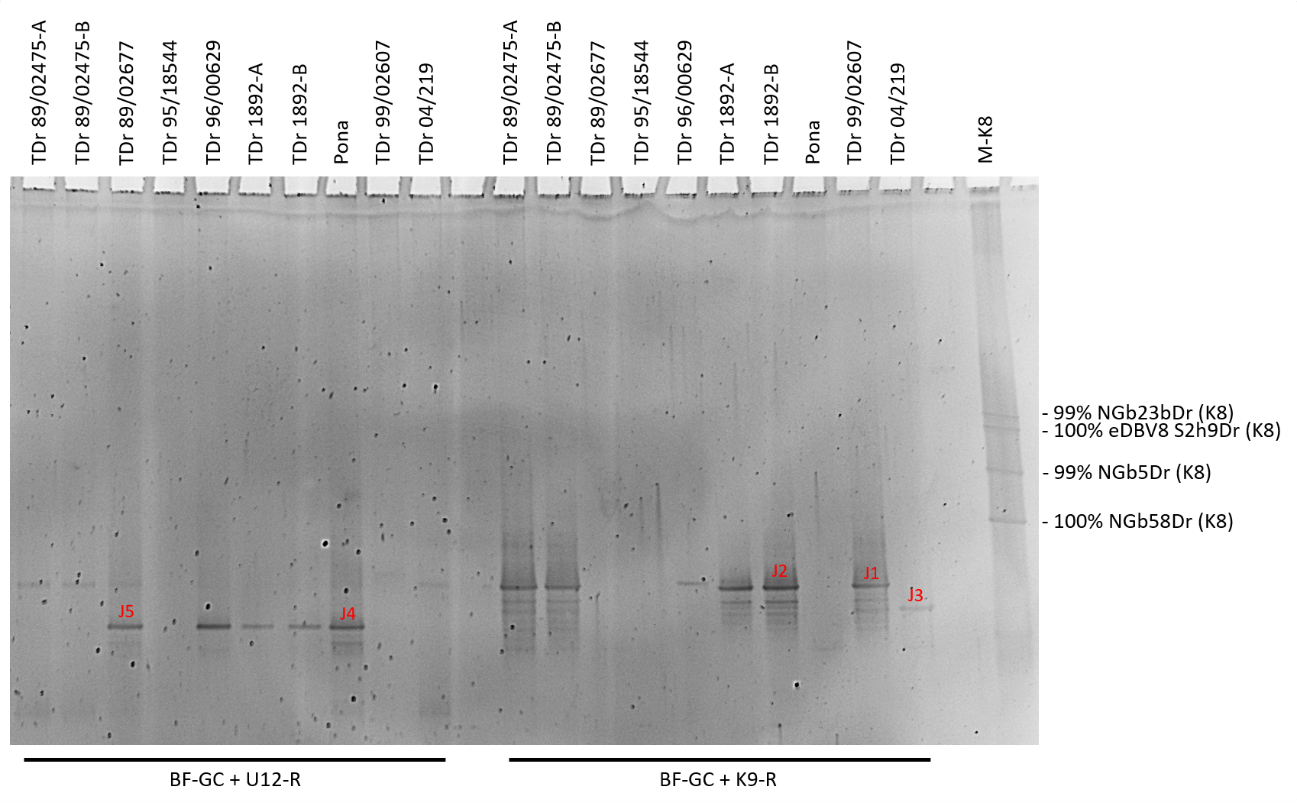
**

**Supplementary Figure 2.** Group K9 versus group U12 single plex PCR-DGGE analysis of partial RT-RNaseH badnavirus sequences from one landrace (Pona) and nine breeding lines (TDr) of *D. rotundata*. Single plex PCR were done with clamp primer BF-GC as forward primer and then either U12-R or K9-R. *D. rotundata* accession TDr 89/02475-A and -B and TDr 1892-A and -B are clones of the same yam accessions. *D. rotundata* breeding lines TDr 99/02607 and TDr 04/219 were included for comparison. Five distinct bands (J1 – J5) were excised, re-amplified, cloned and sequenced. Typically, two clones per excised DGGE band were sequenced, and both sequences are presented in Table 1 except for those which were found to be 100% identical to each other.

**
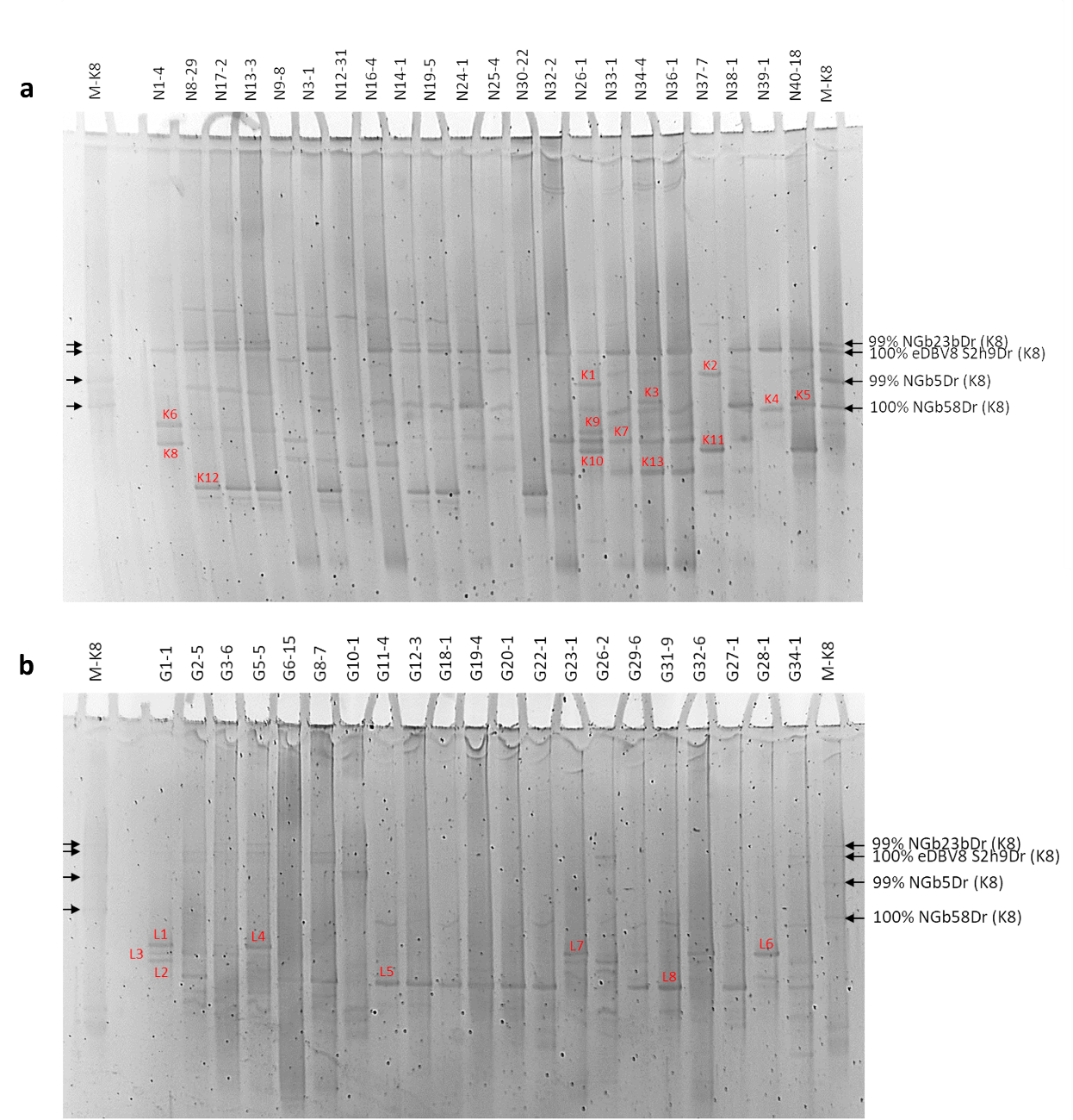
**

**Supplementary Figure 3**. Multiplex PCR-DGGE analysis of yam field samples from Nigeria (a) and Ghana (b). M-K8 denotes DGGE sequence marker (see section 3.6). Twenty one distinct bands (K1 – K13; L1 – 8) were excised, re-amplified, cloned and sequenced. Typically, two clones per excised DGGE band were sequenced, and both sequences are presented in Table 1 except for those which were found to be 100% identical to each other.

## Supplementary Tables

**Table S1**. Primers used for multiplex Badna-PCR.

| **Name** | **Sequence (5'-3')** | **Location^a^** | **Amplicon size (bp)** |
| --- | --- | --- | --- |
| K8-F | CAAAGAACATGGGCTGGT | 138-155 | 415^b^ |
| K8-R | ATACCAAGCCATGACC | 305-320 | 347^c^ |
| K9-R | GAGTATAAAGGGCCAAGTATT | 360-380 | 407^c^ |
| U12-R | GTAGGCGTCTTCAGGGGG | 481-498 | 525^c^ |
| ^a^ Position in 528 bp long partial RT-RNAseH region | | |  |
| ^b^ In combination with Badna-RP | |  |  |
| ^c^ In combination with Badna-FP | |  |  |

Design rationale of new primers in Table S1: The majority of DBV sequences previously identified by DGGE belong to yam badnavirus monophyletic species group K8, followed by groups K9 and U12 (Turaki et al., 2017) and all three species groups have been shown to contain eDBV sequences (Umber et al., 2014). Hence, we focused our attention on these three species groups. Species group K8 was targeted first as it has been found the most diverse DBV species group by Kenyon et al. (2008), Bousalem et al. (2009) and Seal et al. (2014). A total of 204 partial RT-RNaseH sequences available from GenBank belonging to group K8 and containing a mixture of episomal DBV and eDBV sequences were aligned and searched for conserved regions inside (nested) the Badna-FP/-RP binding sites (Yang et al., 2003). The aim was to design a group K8-specific forward or reverse primer, which could be combined with either the Badna-FP or Badna-RP generic primer in PCR amplifications targeting a large enough sequence stretch of the partial RT-RNaseH region enabling taxonomic assessment of badnaviruses (King et al., 2012). Two potential group K8-specific primer sites, one forward (K8-F) and one reverse (K8-R) primer (Table S1), were identified in the consensus sequence of the group K8 alignment PCR amplifications using K8-F in combination with Badna-RP produced amplicons of 415 bp, whereas K8-R in combination with Badna-FP lead to products of 347 bp in length.

Species-specific K9 and U12 reverse primers were designed to be used in a multiplex PCR in combination with generic badnavirus and K8 specific primers. A total of 73 and 34 partial RT-RNaseH sequences available from GenBank belonging to group K9 and U12, respectively, were aligned and searched for conserved regions inside the Badna-FP/-RP binding sites. One primer site for each of the two groups was identified in the respective consensus sequences. Primers K9-R and U12-R are located at position 360-380 and 481-498, respectively, of the 528 bp long partial RT-RNaseH sequence (Table S1). PCR amplifications using K9-R or U12-R in combination with Badna-FP produced amplicons of 407 bp and 525 bp, respectively.

**Table S2**. Yam field samples from Nigeria and Ghana

**Table S3**. High resolution melt cluster analysis of DGGE clones.
